# The Use of a Powder Obtained from Rosehip Waste to Reformulate Pork Sausages: Impacts on Their Quality

**DOI:** 10.3390/foods14061067

**Published:** 2025-03-20

**Authors:** Alexandra Raluca Borşa (Bogdan), Melinda Fogarasi, Floricuța Ranga, Andrei Borșa, Anda Elena Tanislav, Vlad Mureșan, Cristina Anamaria Semeniuc

**Affiliations:** 1 Department of Food Engineering, University of Agricultural Sciences and Veterinary Medicine of Cluj-Napoca, 3-5 Calea Mănăştur, 400372 Cluj-Napoca, Romania; raluca.borsa@usamvcluj.ro (A.R.B.); melinda.fogarasi@usamvcluj.ro (M.F.); andrei.borsa@usamvcluj.ro (A.B.); anda.tanislav@usamvcluj.ro (A.E.T.); vlad.muresan@usamvcluj.ro (V.M.); 2 Department of Food Science, University of Agricultural Sciences and Veterinary Medicine of Cluj-Napoca, 3-5 Calea Mănăştur, 400372 Cluj-Napoca, Romania; floricutza_ro@yahoo.com

**Keywords:** physicochemical properties, colour properties, texture properties, sensory properties, polyphenolic compounds, carotenoid compounds

## Abstract

The powder obtained from rosehip waste can be used as an ingredient in meat products because it contains polyphenolic compounds with preservative and antioxidant effects and carotenoid compounds with a colouring effect. This study aimed to evaluate how partially replacing raw meat with this powder impacts the quality of pork sausages. Therefore, three sausage formulations (PSc-control pork sausages; PS2.7%rp-pork sausages with 2.7% powder from rosehip waste; PS5.5%rp-pork sausages with 5.5% powder from rosehip waste) were prepared and evaluated during storage from physicochemical, colour, texture, and sensory points of view, as well as for their polyphenol and carotenoid contents. The use of the powder from rosehip waste as an ingredient in pork sausages resulted in a significant decrease in moisture and protein content but an increase in total carbohydrates, polyphenols, and carotenoids; additionally, it significantly decreased the pH and easily hydrolysable nitrogen content of sausages, thus demonstrating a preservative effect. It also positively influenced their colour (by intensifying the shades of red and yellow), as well as the sausages’ gumminess and chewiness (by reducing them). Although it slightly affected their taste and texture, the consumer acceptance rate for reformulated sausages was reasonable. In conclusion, the powder obtained from rosehip waste is a promising functional ingredient in pork sausage reformulation.

## 1. Introduction

Sausages are a category of meat products widely consumed in many countries due to their readiness for consumption without additional processing, specific pleasant taste and aroma, and relatively long shelf life [1]. They contain meat, seasonings, and additives such as nitrates, nitrites, or polyphosphates [2]. Nitrites are synthetic additives commonly used as curing agents in meat products [3]. Because of their reputation of being toxic to human health, consumers are increasingly interested in purchasing products without them [4]. Since nitrites are the main preservatives used in meat products, finding suitable substitutes can be challenging [5]. After the IARC (International Agency for Research on Cancer) classified nitrite-cured meat as a Group 1 carcinogen in 2018, many researchers have sought alternatives to replace nitrites [6].

Some researchers have explored the possibility of using rosehip powder and extract in various meat products. In 2012, Vossen et al. [7] assessed the effect of a rosehip extract rich in polyphenols and ascorbic acid, in combination with or without sodium nitrite, on lipid and protein oxidation, colour stability, and the texture of frankfurters. They concluded that while the extract can serve as a natural antioxidant in frankfurters, it cannot fully replace sodium nitrite. Armenteros et al. [8] also evaluated the effect of a phenolic-rich extract derived from rosehips in frankfurters, with and without sodium ascorbate and sodium nitrite. Their findings indicated that using this extract, in combination with traditional antioxidant additives, improved the oxidative stability of frankfurters without altering their colour and texture properties. İlyasoğlu [9] tested the antioxidant effectiveness of rosehip seed powder in raw and cooked meatballs during refrigerated storage, using concentrations of 1%, 2%, and 4%. Their results showed that this powder may be a natural antioxidant for both types of meatballs if used at concentrations higher than 2%. In the study by Nicorescu et al. [10], the antioxidant efficiency of neutralised polyphenolic compounds extracted from rosehip, sodium nitrite, butylated hydroxyanisole, and tetrasodium pyrophosphate in smoked sausages was compared at various concentrations and combinations. Their results showed that the treatment with 0.005% rosehip extract and 0.01% sodium nitrite offered protection against lipid peroxidation as effectively as the mixture of 0.01% sodium nitrite, 0.005% butylated hydroxyanisole, and 0.3% tetrasodium pyrophosphate. Kenenbay et al. [1] investigated the possibility of using rosehip extract as a natural ingredient in cooked sausages. They tested it at concentrations of 3%, 8%, and 13% of the finished product compared to sodium nitrite. The rosehip extract exhibited antioxidant activity when used at a concentration of 13%. Gwak et al. [11] recently assessed the potential of radish and Chinese cabbage powders as natural sources of sodium nitrite in pork sausages; the radish powder resulted in cured pork sausages with desirable colour and pigment properties, while Chinese cabbage powder was less efficient.

The powder obtained from rosehip waste is rich in fibres, polyphenols, and carotenoids; a recent study used it to partially replace wheat flour in a waffle cone recipe, enriching them with these compounds [12]. Due to polyphenols’ preservative and antioxidant effects [13], carotenoids’ colouring effect [14], and this powder’s acidic pH [12], it could be used as an ingredient in meat products to avoid the use of nitrites as synthetic additives or to extend the shelf life. According to Regulation (EC) No. 1333/2008 [15], the maximum amount of nitrite ion (NO_2_) permitted to be added to sausages during their manufacturing is 100 mg/kg, while the maximum residual amount of nitrite ion (NO_2_) in them throughout their shelf life must not exceed 50 mg/kg. Nitrites are usually added to meat products to improve their organoleptic characteristics, inhibit microorganism growth, and prevent toxin production [16]. They contribute to the characteristic cured flavour of these products, stabilise their red colour, and protect against lipid oxidation. Nitrites also show important bacteriostatic and bactericidal activity against several spoilage bacteria and foodborne pathogens [17]. During processing, they can react with amines in meat, generating nitrosamines. These compounds are highly toxic and have been linked to several human diseases, such as liver, colon, oesophageal, and gastric cancer [18].

According to Article 6 (1) and (2) of the Waste Framework Directive [19], certain specified waste ceases to be waste when it has undergone a recovery operation (including recycling) and complies with specific criteria, in particular when (1) the substance or object is commonly used for specific purposes, (2) there is an existing market or demand for the substance or object, (3) the use is lawful (substance or object fulfils the technical requirements for the particular purposes and meets the existing legislation and standards applicable to products), and (4) the use will not lead to overall adverse environmental or human health impacts. Considering these end-of-waste criteria, rosehip powder can be regarded as a byproduct because it is prepared from the waste generated by processing rosehip fruits into purée to be reused as an ingredient in a meat product.

This study aimed to investigate the technological potential of a powder prepared from rosehip waste for applications in the meat industry; to our knowledge, it is the first to use such an ingredient in pork sausages by partially replacing the raw meat. The experimental design followed the impact of this powder on the quality of reformulated pork sausages by evaluating their physicochemical, textural, and sensory properties, as well as their colour and content of polyphenolic and carotenoid compounds. In this regard, three pork sausage formulations were prepared: one control (without the powder obtained from rosehip waste), one containing 2.7% powder from rosehip waste, and another with 5.5% powder from rosehip waste. All formulations were kept at 4 °C for 26 days (the shelf life of control pork sausages) and analysed on days 1, 12, 19, and 26 of storage.

## 2. Materials and Methods

### 2.1. Preparation of Powder from Rosehip Waste

Twelve kilograms of rosehips from the *Rosa canina* L. species, collected from Romanian spontaneous flora (Aiud and Blaj), was acquired in October 2023 from the National Forest Administration Romsilva, the Alba Forestry Department. They were sorted and cleaned (to remove spoiled/rotten fruits and physical impurities), washed with tap water and strained, air-dried (to remove excess water), and finally weighed. Next, they were cold pressed using an electric oil press machine (PU05; S.C. Jobs Ahead Group S.R.L., Bucharest, Romania) to obtain the rosehip purée; the resulting residue was used to prepare the powder, as described below, with a pressing yield of 66%.

The rosehip waste was dried for 24 h at 40 °C using a dehydrator (DEH-450; Biovita S.R.L., Cluj-Napoca, Romania), with a dehydration yield of 28%. Subsequently, it was subjected to sieving to remove allergenic debris and then ground to a fine powder (<10 μm) with an electric grinder (GR-020; Minimoka, Paris, France). The powder thus obtained was packed in hermetically sealed glass jars and stored in a refrigerator, in the dark, until use.

### 2.2. Preparation of Pork Sausages

Two batches of pork sausages were prepared as described below. Thirty kilograms of pork chunks was used to prepare the cured meat, purchased from a local butchery (S.C. Cina Carmangerie S.R.L., Cluj-Napoca, Romania). They were mixed with 0.6 kg of non-iodised salt (Salrom, Bucharest, Romania), kept at 4 °C for 48 h in a chilling room for ageing, and then were ground through a 5 mm sieve with a wolf machine (Bizerba SE & Co. KG, Balingen, Germany). The cured meat thus obtained was divided into two portions: one of 9.0 kg, which was used to prepare the batter, and the other of 21.0 kg, which was kept for later use in the recipe for manufacturing the pork sausages.

The 9.0 kg portion of cured meat was finely chopped and mixed with 0.105 kg of black pepper (Trumf International S.R.O., Dolní Újezd, Czech Republic), 0.090 kg of sweet paprika (Trumf International S.R.O., Dolní Újezd, Czech Republic), 0.030 kg of nutmeg (Trumf International S.R.O., Dolní Újezd, Czech Republic), 0.075 kg of garlic granules (S.C. Ion Moș S.R.L., Chiajna, Romania), 0.090 kg of polyphosphates (Brätfix; Frutarom Savory Solutions, Salzburg, Austria), and 2.7 kg of ice flakes in a meat cutter (Meprotec GmbH, Pasching, Austria), to prepare the batter.

Three formulations of pork sausages were prepared:

1. The control sample (PSc);
2. The sample with 2.7% powder from rosehip waste (PS2.7%rp, resulting from replacing 3% of the cured meat weight with the powder);
3. The sample with 5.5% powder from rosehip waste (PS5.5%rp, resulting from replacing 6% of the cured meat weight with the powder).

The remaining cured meat was mixed with the batter and powder from rosehip waste (except for the control sample, PSc) according to the manufacturing recipes in Table 1. The mixture thus obtained was inserted into collagen casings (S.C. Darimex International S.R.L., Otopeni, Romania) using a vacuum filling machine (Düker-REX Fleischereimaschinen GmbH, Laufach, Germany), the sausages being portioned and linked manually by twisting at 20–25 cm intervals. The sausage bars thus formed were hung on stainless-steel sticks placed on a rack and then introduced into a boiling–smoking cell, where they were subjected to the following treatments: air-drying (45–75 °C/30 min), hot smoking (75 °C/30 min), and cooking (74 °C/40 min); they were then left to cool at room temperature (approximately 60 min), after which they were stored in a chilling room at 4 °C for 26 days. Samples were taken on storage days 1, 12, 19, and 26 to be analysed as described below.

For each formulation, 9.7 kg of pork sausages was obtained per batch; one kilogram of PSc, PS2.7%rp, and PS5.5%rp from each batch was frozen at −18 °C immediately after preparation and kept in the freezer until sensory analysis.

### 2.3. Analysis Methods

Pork sausages were tested for proximate composition, including moisture, protein, fat, crude fibre, and ash content, using the methods published in ISO 1442:2023 [20], ISO 937:2023 [21], ISO 1443:1973 [22], ISO 5498:1981 [23], and ISO 936:1998 [24] standards. The total carbohydrate content (%) was calculated as 100 − (% moisture + % protein + % fat + % ash), and the energy value (kcal/100 g) was computed as 4 × (g protein + g carbohydrate) + 9 × g fat according to Fogarasi et al. [25]. Using a digital multi-parameter meter (InoLab Multi 9310 IDS; WTW, Weilheim, Germany), the pH of the aqueous extract of pork sausages was also measured, as Socaciu et al. [26] described. The easily hydrolysable nitrogen (EHN) in pork sausages was determined using the method from SR 9065-7:2007 standard [27]. Each batch’s analyses were duplicated, so there were four measurements.

The NH300 colorimeter (3NH, Shenzhen, China; illuminant: D65; measuring aperture: Φ 8 mm; standard observer: 10°) was used to measure the colour of pork sausages based on the CIE L*a*b* colour scheme, as described by Socaciu et al. [28]. Three slices (1 cm thick) from every batch of pork sausages were used for this determination; 2 measurements were taken on each slice, so 12 measurements in total.

The texture profile of pork sausages was analysed using a CT3 texture analyser (Brookfield Engineering Laboratories Inc., Middleboro, MA, USA). Each sausage sample (20 mm in height and 25 mm in diameter) was consecutively taken from the fridge and submitted to a two-cycle compression. The test involved sample deformation to 30% of its surface, using a TA4/1000 cylindrical probe (20 mm long and 38.1 mm in diameter) attached to a 10 kg compression cell, with a speed of 1 mm/s. The parameters recorded by the software used (TexturePro CT software) were hardness cycle 1 (N), hardness work cycle 1 (mJ), hardness cycle 2 (N), hardness work cycle 2 (mJ), cohesiveness, springiness index, gumminess (N), and chewiness (mJ). The samples from days 19 and 26 of storage were analysed using the TA39 cylinder probe (20 mm long and 2 mm in diameter); they were harder and had significantly smaller diameters due to water losses. The same test was applied to the overturned sample to determine the surface hardness. Two measurements were taken for each batch; therefore, it was quadruplicated.

The hedonic and purchase intention tests were applied to evaluate the sensory attributes of the pork sausage formulations; the Bioethics Committee of the UASVM (University of Agricultural Sciences and Veterinary Medicine of Cluj-Napoca) approved the sensory testing protocol (No. 441 from 17 April 2024). The sensory analysis of sausage samples was conducted in the Sensory Analysis Laboratory within the Faculty of Food Science and Technology with volunteers (students, master’s students, PhD students, teaching staff, researchers, and other employees of UASVM).

In total, 86 volunteers (58 female and 28 male), with an average age of 27, rated the pork sausages in terms of liking their appearance, colour, odour, taste, texture, and overall acceptability (from dislike extremely (1) to like extremely (9)). The overall score of each formulation was calculated as the average of scores received following this assessment; the acceptance rate was calculated by dividing the overall score by the maximum score obtained by a formulation and multiplying it by 100. This study’s participants also expressed their willingness to buy the pork sausages (by giving marks from 1 (definitely will buy) to 5 (definitely will not buy)).

The pork sausages were first thawed (kept in the refrigerator from evening to morning), grilled (using programme 3 of Super Grill G01-M; Tefal, Hong Kong, China) for 2.5 min on each side, and finally cut into slices of 2 cm in thickness. Two slices of each formulation were placed on a preheated white porcelain dish (marked with three-digit sample numbers) and then covered with aluminium foil to be served hot. The sensory session was conducted in individual cabins at a controlled temperature (20 °C) under natural light. Participants were advised not to drink, eat, or smoke for at least one hour before testing the sausage samples. They were also asked to cleanse their palate by eating a piece of bread before evaluating another formulation.

Polyphenolic compounds were extracted from a sample (0.5 g of powder and 5.0 g of sausage) with a methanol/water mixture [70:30 (*v*/*v*); 5 mL] to quantify them in pork sausages, as detailed by Fogarasi et al. [25]. An Agilent 1200 HPLC system (Palo Alto, CA, USA) was employed to perform the chromatographic analysis of the resulting extracts by applying the HPLC-DAD-ESI/MS method of Fogarasi et al. [29].

The content of carotenoid compounds in powder from rosehip waste and pork sausages was estimated by extracting them from a sample (1.0 g) using a methanol/ethyl acetate/petroleum ether mixture [1:1:1 (*v*/*v*/*v*); 10 mL], as proposed by Szabo et al. [30]. The resulting extracts were then chromatographically analysed using their HPLC-DAD method on the same HPLC equipment.

### 2.4. Statistical Analysis

One-way ANOVA with Tuckey’s post hoc test was used to evaluate the effect of partially replacing cured ground meat with powder from rosehip waste and of storage time on qualitative parameters of pork sausages, with a 95% confidence level (*p* < 0.05). Minitab statistical software (version 19.1.1; LEAD Technologies, Inc., Charlotte, NC, USA) was used for this analysis.

## 3. Results and Discussion

### 3.1. Physicochemical Properties of Powder from Rosehip Waste and Pork Sausages

The powder from rosehip waste, prepared as described above (Section 2.1), was characterised by a content of 11.46 ± 0.195% moisture, 6.86 ± 0.100% protein, 3.62 ± 0.060% fat, 56.39 ± 1.087% total carbohydrates (of which 44.09 ± 0.830% was crude fibre), and 2.52 ± 0.012% ash, an energy value of 286 ± 5.303 kcal/100 g, and a pH of 4.02 ± 0.007. These findings are comparable to those of Borşa et al.’s study [12] regarding the proximate composition of their powder from rosehip waste.

**Figure 1:**
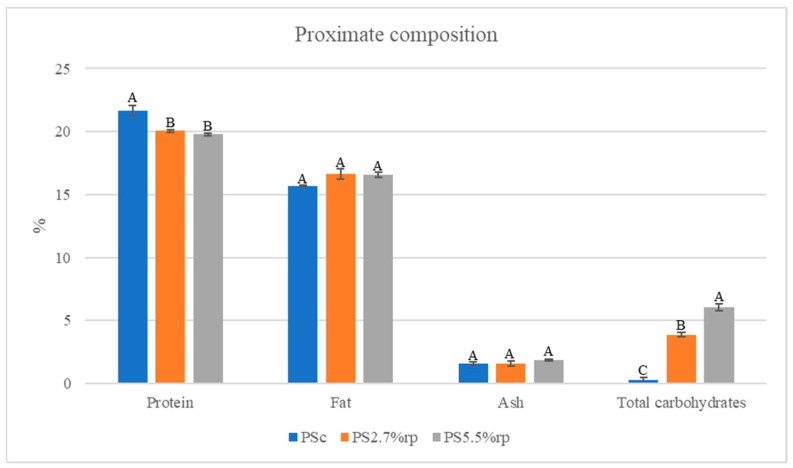
Proximate composition of pork sausage formulations. Data are expressed as mean ± standard deviation values of four measurements. Different uppercase letters indicate significant differences between pork sausage formulations (*p* < 0.05, Tukey’s test).

Figure 1 and Table 2 show the results regarding the proximate composition of pork sausage formulations. Replacing raw meat with the powder from rosehip waste resulted in a significant decrease in the moisture content of pork sausages (from 60.83% in PSc to 57.83% in PS2.7%rp, respectively, to 55.79% in PS5.5%rp) and, consequently, an increase in their total dry matter content and energy value (from 229 ± 0.138% ^B^ in PSc to 245 ± 3.758% ^A^ in PS2.7%rp, respectively, to 252 ± 3.401% ^A^ in PS5.5%rp; the different uppercase letters indicate significant differences between pork sausage formulations (*p* < 0.05, Tukey’s test). These findings align with previous studies, which showed lower moisture content in sausages made with vegetable powders (such as beetroot and radish [31], cherry [32], and tomato byproducts [2]) than the control sausages.

The moisture content of pork sausages decreased significantly during the 26 days of storage, particularly in those formulated with powder from rosehip waste, reaching 37.61% in PSc, 27.97% in PS2.7%rp, and 21.49% in PS5.5%rp (Table 2); this could be due to the loss of hydrogen-bonded water molecules caused by the interaction of myofibrillar proteins and polyphenols, which reduced the meat proteins’ water-holding capacity [32]. The higher total dry matter content in reformulated sausages was due to the significant increase in their total carbohydrate content with the amount of powder used as an ingredient (from 0.25% in PSc to 3.86% in PS2.7%rp, respectively, to 6.06% in PS5.5%rp); in contrast, the protein content decreased (from 21.68% in the control sample to 20.06–19.77% in the samples with powder). However, the content of fat (15.69–16.54%) and ash (1.56–1.85%) in pork sausages was not significantly affected. As the powder obtained from rosehip waste is rich in carbohydrates, a similar effect of increasing their content was also reported by Borşa et al. [12] in waffle cones when this ingredient was used to enrich them with fibre.

Given the acidic pH of the powder from rosehip waste, it significantly decreased the pH of pork sausages from 6.13 in PSc to 5.97 in PS2.7%rp, respectively, to 5.68 in PS5.5%rp (Table 2). A similar pH-decreasing effect with the amount of powder from rosehip waste used as an ingredient was also noticed by Borşa et al. [12] in enriched waffle cones. In addition, the organic acids present in the powder neutralised the ammonia from the raw meat, leading to a significant decrease in the easily hydrolysable nitrogen content, an indicator of meat product freshness: from 24.61 mg NH_3_/100 g in PSc to 21.18 mg NH_3_/100 g in PS2.7%rp, respectively, to 16.92 mg NH_3_/100 g in PS5.5%rp.

During storage, the pH significantly increased in PSc (to 6.23) but decreased in PS2.7%rp (to 5.84) and PS5.5%rp (to 5.60), thus demonstrating the preservative effect of powder from rosehip waste; however, it significantly increased during storage in all three sausage formulations (reaching 33.14 mg NH_3_/100 g in PSc, 33.12 mg NH_3_/100 g in PS2.7%rp, and 29.74 mg NH_3_/100 g in PS5.5%rp). These findings correlate with the pH results, indicating that the observed changes are due to the water losses of sausage samples rather than the accumulation of ammonia through the breakdown of meat proteins [25]. Moreover, the content of easily hydrolysable nitrogen was well below the maximum allowed limit (45 mg NH_3_/100 g) [33] in all sausage samples at all storage times.

### 3.2. Colour Properties of Powder from Rosehip Waste and Pork Sausages

The colorimetric measurements revealed the following values for powder from rosehip waste: 62.08 ± 1.745 for L*, 17.40 ± 0.501 for a*, 32.72 ± 1.227 for b*, 1.08 ± 0.015 for h*, and 37.06 ± 1.205 for C*; these results indicate a moderately intense red-orange colour for the powder, like the findings of Borşa et al. [12]. Its use as an ingredient in formulating pork sausages determined a “large” total colour difference between the sample containing 2.7% powder and the control one (ΔE* = 6.2) but “obvious” between the one with 5.5% powder and the control sample (ΔE* = 13.3) (Table 3). This discrepancy is attributed to significant changes in several colour attributes, including an increase in the b*-value (yellowness) for PS2.7%rp, respectively, and a decrease in the L*-value (lightness) along with increases in the a*-value (redness) and b*-value (yellowness) for PS5.5%rp. The calculated values for h* (the dominant colour) and C* (the colour intensity) showed that the sausages’ colour shifted towards orange and intensified with the increasing concentration of powder from rosehip waste in their formula. Igual et al. [34] reported similar trends for the colour attributes of corn extrudates in a study that used rosehip powder to enrich them. In the study by Borşa et al. [12], using powder from rosehip waste as an ingredient in waffle cones significantly reduced their lightness and yellow shade intensity; however, it did not affect their red shade, likely due to the baking process involved in their preparation.

The colour of pork sausage formulations changed during storage due to their concentrating (by dehydration) and oxidation of pigments. The higher water losses during storage in the reformulated sausages compared to the control ones determined an increase in their ΔE*-values on the last day of storage to 19.5 in PS2.7%rp and 19.8 in PS5.5%rp. The lightness of the pork sausages decreased with storage time in all three formulations. The red shade of pork sausages faded by the 19th day of storage due to the oxidation of myoglobin and haemoglobin in the raw meat [26]; after this point, the redness intensified until day 26. As for the yellow shade, it remained relatively stable until the 19th day of storage, when it intensified.

### 3.3. Texture and Sensory Properties of Pork Sausages

The instrumental measurement results of texture attributes for the three sausage formulations during storage are summarised in Table 4. Replacing pork meat with powder from rosehip waste slightly decreased the sausages’ gumminess (at 51.79–54.31 N) and chewiness (at 293.70–285.07 mJ) from values of 63.51 N and 368.23 mJ, respectively; however, their surface hardness (23.22–23.92 N), hardness (73.79–83.45 N), cohesiveness (0.68–0.76), and springiness index (0.80–0.86) were not significantly influenced. These results share a few similarities with Duggirala et al.’s [35] findings, which showed a significant increase in the hardness of raw ground beef patties when formulated with 1–3% rosehip powder, with the other texture parameters varying not significantly. Unlike us, Gül and Şen [36] reported a significant decrease in the hardness and cohesiveness of bread formulated with 5% rosehip powder but an increase in the gumminess. Furthermore, Gurbuz and Demirci [37] noted a significant decrease in the cohesiveness of yoghurt fortified with 1–3% rosehip powder.

The product’s geometry changed during storage due to shrinkage caused by water loss. For this reason, a cylinder sample of smaller diameter was necessary to analyse the texture profile of sausage samples from day 19 of storage onwards. Consequently, the effect of storage time on the sausages’ textural properties was discussed by comparing the results of samples from days 1 and 12, respectively, from days 19 and 26.

Surface hardness, hardness, gumminess, and chewiness were the textural attributes of pork sausage most affected by storage. Surface hardness increased significantly until the 12th day of storage for all three sausage formulations and from day 19 to day 26 only for the formulations with powder from rosehip waste. In terms of hardness, it significantly varied between days 1 and 12 of storage only for the PS5.5%rp sample, while between days 19 and 26 it increased for all samples. Gumminess, however, increased significantly with storage time only in the reformulated sausages case, and a similar trend was observed for chewiness. In contrast to our results, Duggirala et al. [35] reported a decrease in the hardness of raw ground beef patties incorporated with rosehip powder during 7 days of storage at 4 °C.

Table 5 and Table 6 summarise the sensory analysis results of the three sausage formulations. The PS2.7%rp sample’s acceptance rate was similar to that of the control sausages (86%), but it was lower for the PS5.5%rp sample (83%) (Table 5); this is due to the significant reduction in hedonic scores obtained by the reformulated sausages for texture (from 8.0 in PSc to 7.4 in PS2.7%rp and 7.1 in PS5.5%rp) and taste (from 8.0 in PSc to 7.6 in PS2.7%rp and 7.4 in PS5.5%rp) with the amount of powder from rosehip waste used as an ingredient, even though those for colour significantly rose (from 7.3 in PSc to 7.7 in PS2.7%rp and 7.8 in PS5.5%rp). The other sensory attributes evaluated, such as appearance (7.5–7.6), odour (7.9–8.0), and overall acceptability (7.6–7.8), were not significantly influenced by the powder from rosehip waste; consequently, neither was the overall score (7.5–7.7), which ranged between “like moderately” and “like very much”. Given that an acceptance rate ≥ 70% is reasonable [25], either reformulated sausage sample can be launched on the market. Vartolomei and Turtoi [38] instead reported higher acceptance rates (90.5–94.5%) for bread formulated with 0.5–2.5% rosehip powder and 88.5% for the control one. In the study by Popovici et al. [39], the incorporation of rosehip powder as a functional ingredient in cashew-based candies caused a depreciation of their external appearance at a concentration of 5%, as well as of their smell, taste, and consistency at a concentration of 10%. Chochkov et al. [40] used rosehip powder (5–15%) as a flour replacement ingredient in bread. As the powder concentration increased in the formulation, it negatively affected the bread’s appearance, crumb colour, and porosity; however, it improved the taste, aroma, and crust colour.

Although PS2.7%rp’s acceptance rate coincided with that of PSc, only 24% of panellists answered with “definitely will buy” for this sample (Table 6); 40% expressed the same willingness for the control sample and the remaining 24% for the sample with 5.5% powder from rosehip waste. The percentage of those undecided about buying the reformulated sausages was also higher for PS2.7%rp (17%) and PS5.5%rp (23%) compared to PSc (13%). These findings indicate a reticence among consumers to accept the powder from rosehip waste as an ingredient in pork sausages. Nevertheless, a marketing strategy emphasising this powder’s health benefits and its environmentally friendly aspect of reducing rosehip waste could considerably increase consumer intentions to purchase the reformulated sausages. Given that 44% of the panellists responded with “probably will buy” for the sausages with 2.7% powder from rosehip waste and only 24% for those with 5.5% powder, the PS2.2%rp sample seems to have the best potential for industrial production.

### 3.4. Content of Polyphenolic and Carotenoid Compounds in Powder from Rosehip Waste and Pork Sausages

The chromatographic determination results of polyphenols and carotenoids in powder from rosehip waste and pork sausages are presented in Table 7 and Table 8. The powder exhibited a high content of polyphenolic compounds (25,007.15 µg/g), of which 17,883.92 µg/g of flavonoids (17,144.18 µg/g of flavanols, 704.05 µg/g of flavonols, and 35.69 µg/g of anthocyanins) and 7123.23 µg/g of phenolic acids (7034.11 µg/g of hydroxybenzoic acids and 89.12 µg/g of hydroxycinnamic acids). Among the 18 polyphenolic compounds identified (Table 7), the most abundant were catechin (5237.86 µg/g), 2-hydroxybenzoic acid (3533.84 µg/g), procyanidin dimer B2 (3500.26 µg/g), procyanidin trimer C2 (3414.31 µg/g), and procyanidin dimer B3 (2433.35 µg/g). Unlike us, Ghendov-Mosanu [41] found in their rosehip powder a polyphenolic profile dominated by procyanidin dimer B1 (2910 µg/g), followed by chlorogenic acid (1050 µg/g); the other 15 compounds they identified were minor (0.1–570 µg/g).

Only two polyphenolic compounds, 2-hydroxybenzoic acid (225.51 µg/g) and protocatechuic acid (8.35 µg/g), were found in the control sausages. These compounds came from the spices used in their preparation, determining a total polyphenol content in PSc of 233.85 µg/g (all phenolic acids). The use of powder from rosehip waste as an ingredient in pork sausages had an enrichment effect on them with polyphenols, as 18 such compounds were found in the reformulated samples; their total polyphenolic content was 1.75 times higher in PS2.7%rp (410.62 µg/g, of which 145.95 µg/g was flavonoids and 264.67 µg/g was phenolic acids) and 3.69 times higher in PS5.5%rp (863.69 µg/g, of which 590.37 µg/g was flavonoids and 273.32 µg/g was phenolic acids) than in PSc.

As for the carotenoid compounds, a total of 118.63 µg/g was quantified in the powder from rosehip waste, of which 2.10 µg/g was lutein, 38.74 µg/g was lycopene, and 77.79 µg/g was *β*-carotene (Table 8). In contrast to our findings, Ghendov-Mosanu et al. [41] did not find lutein in their rosehip powder; they reported a content of 3.80 µg/g of *cis*-lycopene, 27.20 µg/g of all-*trans*-lycopene, 4.50 µg/g of *cis*-*β*-carotene, and 18.40 µg/g of all-*trans*-*β*-carotene instead. None of the carotenoid compounds in the powder from rosehip waste were detected in the control sausages; only lycopene and *β*-carotene were found in the reformulated sausages, with lutein likely being below the detection limit. The content of lycopene and *β*-carotene in pork sausages significantly increased with the amount of powder from rosehip waste used in their formulation, reaching a total of 5.02 µg/g in PS2.7%rp and 9.98 µg/g in PS5.5%rp.

## 4. Conclusions

This study demonstrated the technological potential of powder from rosehip waste by using it as an ingredient in pork sausages, partially replacing raw meat. The powder positively impacted the sausages’ physicochemical properties by enriching them with crude fibre and lowering their pH, thus having a preservative effect. Its orange-red colour contributed to maintaining the colour of the reformulated sausages during their manufacturing, and it was less affected by heat treatment than the colour of control sausages. At the same time, the powder from rosehip waste improved the textural attributes of pork sausages by reducing their gumminess and chewiness. From a sensory point of view, it affected the taste and texture of the final product but enhanced its visual appeal through colour. When used in 2.7% and 5.5% concentrations, the powder from rosehip waste enriched pork sausages with polyphenols and carotenoids. In conclusion, valorising rosehip waste as a food powder offers a valuable ingredient for meat products. In addition, it plays a role in minimising food waste and promoting sustainable development in the food industry.

## Figures and Tables

**Table 1:** Ingredients used to formulate the pork sausages.

Ingredient	PSc		PS2.7%rp		PS5.5%rp	
	kg	%	kg	%	kg	%
Cured ground meat	7.0	63.3	6.7	60.9	6.4	58.2
Powder from rosehip waste	-	-	0.3	2.7	0.6	5.5
Batter	4.0	36.4	4.0	36.4	4.0	36.4
Total	11.0	100.0	11.0	100.0	11.0	100.0

**Table 2:** Changes in proximate composition, energy value, pH, and easily hydrolysable nitrogen content of pork sausages during refrigerated storage.

Parameter/ Energy Value	PSc				PS2.7%rp				PS5.5%rp			
	1st Storage Day	12th Storage Day	19th Storage Day	26th Storage Day	1st Storage Day	12th Storage Day	19th Storage Day	26th Storage Day	1st Storage Day	12th Storage Day	19th Storage Day	26th Storage Day
Moisture (%)	60.83 ± 0.21 ^aA^	49.34 ± 0.03 ^bA^	40.01 ± 0.21 ^cA^	37.61 ± 0.51 ^dA^	57.83 ± 0.25 ^aB^	41.29 ± 0.30 ^bB^	37.28 ± 0.30 ^cB^	27.97 ± 0.07 ^dB^	55.79 ± 0.65 ^aC^	39.73 ± 0.43 ^bC^	27.20 ± 0.12 ^cC^	21.49 ± 0.16 ^dC^
pH	6.13 ± 0.01 ^cA^	6.15 ± 0.01 ^cA^	6.20 ± 0.0 ^bA^	6.23 ± 0.01 ^aA^	5.97 ± 0.01 ^aB^	5.97 ± 0.01 ^aB^	5.96 ± 0.01 ^aB^	5.84 ± 0.0 ^bB^	5.68 ± 0.0 ^bC^	5.70 ± 0.0 ^aC^	5.69 ± 0.01 ^bC^	5.60 ± 0.0 ^cC^
EHN (mg NH_3_/100 g)	24.61 ± 1.22 ^bA^	31.44 ± 1.21 ^aA^	31.44 ± 1.20 ^aA^	33.14 ± 1.20 ^aA^	21.18 ± 1.13 ^bA^	31.44 ± 1.20 ^aA^	32.29 ± 2.40 ^aA^	33.12 ± 1.20 ^aA^	16.92 ± 0.08 ^dB^	27.19 ± 0.0 ^cB^	33.98 ± 0.01 ^aA^	29.74 ± 1.21 ^bA^

Data are expressed as mean ± standard deviation values of four measurements. Different lowercase letters within a row indicate significant differences between storage times (*p* < 0.05, Tukey’s test), and different uppercase letters show significant differences between pork sausage formulations (*p* < 0.05).

**Table 3:** Changes in colour properties of pork sausages during refrigerated storage.

Colour Attribute	PSc				PS2.7%rp				PS5.5%rp			
	1st Storage Day	12th Storage Day	19th Storage Day	26th Storage Day	1st Storage Day	12th Storage Day	19th Storage Day	26th Storage Day	1st Storage Day	12th Storage Day	19th Storage Day	26th Storage Day
	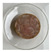	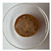	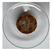	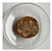	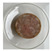	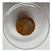	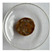	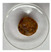	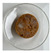	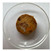	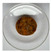	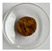
L*	60.15 ± 2.11 ^aA^	52.58 ± 3.89 ^bA^	46.23 ± 2.43 ^cA^	52.28 ± 1.39 ^bA^	59.49 ± 1.42 ^aA^	48.15 ± 3.15 ^bB^	47.10 ± 2.57 ^bA^	40.80 ± 4.95 ^cB^	54.91 ± 1.63 ^aB^	46.92 ± 3.09 ^bB^	42.84 ± 2.64 ^cB^	41.48 ± 3.20 ^cB^
a*	10.01 ± 1.11 ^aB^	7.39 ± 0.72 ^bC^	6.15 ± 0.61 ^cC^	7.43 ± 0.82 ^bB^	10.61 ± 1.73 ^bAB^	8.40 ± 0.73 ^cB^	8.07 ± 1.21 ^cB^	12.71 ± 1.98 ^aA^	11.82 ± 1.29 ^abA^	10.00 ± 0.93 ^cA^	10.41 ± 1.37 ^bcA^	13.40 ± 2.11 ^aA^
b*	10.24 ± 0.69 ^bC^	10.72 ± 1.29 ^abB^	9.50 ± 1.41 ^bC^	11.83 ± 1.07 ^aB^	15.63 ± 3.13 ^bB^	14.38 ± 0.98 ^bA^	15.16 ± 2.37 ^bB^	24.89 ± 6.68 ^aA^	22.06 ± 2.36 ^abA^	15.07 ± 3.26 ^cA^	19.84 ± 4.53 ^bA^	26.28 ± 5.82 ^aA^
h*	0.77 ± 0.08 ^bC^	0.97 ± 0.05 ^aB^	0.99 ± 0.03 ^aB^	1.01 ± 0.03 ^aB^	0.99 ± 0.08 ^bB^	1.04 ± 0.02 ^abA^	1.08 ± 0.02 ^aA^	1.09 ± 0.06 ^aA^	1.08 ± 0.05 ^aA^	0.97 ± 0.08 ^bB^	1.08 ± 0.07 ^aA^	1.09 ± 0.04 ^aA^
C*	14.79 ± 1.43 ^aC^	13.04 ± 1.32 ^bB^	11.32 ± 1.49 ^cC^	13.98 ± 1.29 ^abB^	18.62 ± 2.981 ^bB^	16.66 ± 1.17 ^bA^	17.18 ± 2.63 ^bB^	27.99 ± 6.77 ^aA^	25.06 ± 2.41 ^abA^	18.14 ± 3.06 ^cA^	22.45 ± 4.51 ^bcA^	29.52 ± 6.08 ^aA^
ΔE*	-	-	-	-	6.21	7.01	7.61	19.54	13.33	8.67	12.76	19.83

Data are expressed as mean ± standard deviation values of twenty-four measurements. Different lowercase letters within a row indicate significant differences between storage times (*p* < 0.05, Tukey’s test), and different uppercase letters show significant differences between pork sausage formulations (*p* < 0.05); ΔE*  =  0.0–0.5, a colour difference at the trace level; ΔE*  >  0.5 ≤ 1.5, the colour difference is slight; ΔE*  >  1.5 ≤ 3.0, the colour difference is noticeable; ΔE*  >  3.0 ≤ 6.0, the colour difference is appreciable; ΔE*  >  6.0 ≤ 12.0, the colour difference is large; ΔE*  >  12.0, the colour difference is obvious.

**Table 4:** Changes in texture properties of pork sausages during refrigerated storage.

Texture Attribute	PSc				PS2.7%rp				PS5.5%rp			
	1st Storage Day	12th Storage Day	19th Storage Day	26th Storage Day	1st Storage Day	12th Storage Day	19th Storage Day	26th Storage Day	1st Storage Day	12th Storage Day	19th Storage Day	26th Storage Day
Surface hardness (N)	23.92 ± 1.87 ^bA^	31.43 ± 2.34 ^aC^	19.93 ± 1.15 ^aB^	23.14 ± 2.80 ^aB^	23.22 ± 1.51 ^bA^	54.44 ± 3.44 ^aB^	19.33 ± 1.60 ^bB^	42.76 ± 3.57 ^aA^	22.32 ± 1.80 ^bA^	89.92 ± 2.28 ^aA^	36.79 ± 2.58 ^bA^	45.95 ± 2.34 ^aA^
Hardness (N)	83.45 ± 5.28 ^aA^	93.68 ± 3.97 ^aA^	9.92 ± 0.47 ^bB^	14.40 ± 0.70 ^aB^	73.79 ± 5.80 ^aA^	65.96 ± 5.40 ^aB^	7.84 ± 1.08 ^bB^	29.07 ± 2.26 ^aA^	79.95 ± 5.17 ^bA^	104.93 ± 6.36 ^aA^	18.10 ± 1.71 ^bA^	29.57 ± 4.19 ^aA^
Cohesiveness	0.76 ± 0.03 ^aA^	0.61 ± 0.01 ^bA^	0.36 ± 0.04 ^aAB^	0.42 ± 0.05 ^aA^	0.70 ± 0.01 ^aA^	0.70 ± 0.08 ^aA^	0.60 ± 0.12 ^aA^	0.39 ± 0.01 ^bA^	0.68 ± 0.06 ^aA^	0.66 ± 0.05 ^aA^	0.26 ± 0.14 ^aB^	0.43 ± 0.01 ^aA^
Springiness index	0.85 ± 0.04 ^aA^	0.84 ± 0.02 ^aA^	0.65 ± 0.05 ^bA^	0.94 ± 0.01 ^aA^	0.86 ± 0.03 ^aA^	0.81 ± 0.04 ^aAB^	0.96 ± 0.22 ^aA^	0.92 ± 0.06 ^aA^	0.80 ± 0.03 ^aA^	0.76 ± 0.02 ^aB^	0.51 ± 0.38 ^aA^	0.95 ± 0.05 ^aA^
Gumminess (N)	63.51 ± 6.18 ^aA^	56.73 ± 2.14 ^aAB^	5.20 ± 0.89 ^aA^	4.17 ± 0.48 ^aB^	51.79 ± 3.37 ^aB^	46.24 ± 8.40 ^aB^	4.76 ± 1.40 ^bA^	11.24 ± 0.92 ^aA^	54.31 ± 1.71 ^bAB^	69.41 ± 9.23 ^aA^	4.64 ± 2.16 ^bA^	12.88 ± 1.98 ^aA^
Chewiness (mJ)	368.23 ± 47.18 ^aA^	311.27 ± 23.00 ^aA^	20.10 ± 5.06 ^aA^	27.93 ± 3.32 ^aB^	293.70 ± 15.94 ^AB^	247.80 ± 51.32 ^aA^	27.87 ± 13.40 ^A^	74.77 ± 8.14 ^aA^	285.07 ± 15.22 ^aB^	333.50 ± 37.73 ^aA^	17.20 ± 18.67 ^bA^	86.07 ± 14.95 ^aA^

Data are expressed as mean ± standard deviation values of four measurements. Different lowercase letters within a row indicate significant differences between storage times (*p* < 0.05, Tukey’s test), and different uppercase letters show significant differences between pork sausage formulations (*p* < 0.05).

**Table 5:** Hedonic scores for pork sausage’s sensory attributes and acceptance rates.

Formulation	Sensory Attributes							Acceptance Rate (%)
	Appearance	Colour	Odour	Taste	Texture	Overall Acceptability	Overall Score	
PSc	7.5 ± 1.24 ^A^	7.3 ± 1.37 ^B^	7.9 ± 1.05 ^A^	8.0 ± 1.22 ^A^	8.0 ± 1.11 ^A^	7.8 ± 0.97 ^A^	7.7 ± 0.90 ^A^	86
PS2.7%rp	7.7 ± 1.20 ^A^	7.7 ± 1.14 ^A^	8.0 ± 0.96 ^A^	7.6 ± 1.01 ^AB^	7.4 ± 1.26 ^B^	7.7 ± 1.04 ^A^	7.7 ± 0.76 ^A^	86
PS5.5%rp	7.6 ± 1.49 ^A^	7.8 ± 1.48 ^A^	7.9 ± 1.02 ^A^	7.4 ± 1.47 ^B^	7.1 ± 1.58 ^B^	7.6 ± 1.24 ^A^	7.5 ± 1.14 ^A^	83

Data are expressed as mean ± standard deviation values of eighty-six responses. Different uppercase letters within a column indicate significant differences between pork sausage formulations (*p* < 0.05, Tukey’s test).

**Table 6:** Response rates (%) for purchase intention of pork sausages.

Formulation	Definitely Will Buy	Probably Will Buy	Might or Might Not Buy	Probably Will Not Buy	Definitely Will Not Buy
PSc	40	38	13	6	3
PS2.7%rp	24	44	17	8	6
PS5.5%rp	24	24	23	16	12

**Table 7:** Content of polyphenolic compounds (µg/g) in rosehip waste powder and pork sausages.

Crt. No.	Compound	Class/ Subclass	Powder From Rosehip Waste	PSc	PS2.7%rp	PS5.5%rp
1	2-Hydroxybenzoic acid	PAs/HBAs	3533.84 ± 158.87	225.51 ± 3.19 ^B^	221.06 ± 2.82 ^B^	240.74 ± 0.51 ^A^
2	Procyanidin dimer B1	FVs/FVals	1329.77 ± 126.82	n.d.	16.80 ± 0.09 ^B^	35.40 ± 1.62 ^A^
3	Protocatechuic acid	PAs/HBAs	1239.40 ± 34.49	8.35 ± 1.10 ^C^	12.47 ± 0.33 ^B^	21.10 ± 1.02 ^A^
4	Procyanidin dimer B3	FVs/FVals	2433.35 ± 9.32	n.d.	33.40 ± 0.09 ^A^	38.53 ± 1.09 ^A^
5	Cyanidin 3-*O*-glucoside	FVs/ACNs	35.68 ± 2.43	n.d.	2.68 ± 0.01 ^B^	2.85 ± 0.02 ^A^
6	Procyanidin dimer B2	FVs/FVals	3500.26 ± 241.89	n.d.	22.40 ± 0.28 ^B^	54.31 ± 2.16 ^A^
7	Procyanidin trimer C2	FVs/FVals	3414.31 ± 284.83	n.d.	21.27 ± 0.86 ^A^	35.95 ± 5.04 ^A^
8	Catechin	FVs/FVals	5237.86 ± 274.70	n.d.	22.46 ± 0.09 ^B^	43.44 ± 2.71 ^A^
9	Procyanidin trimer C1	FVs/FVals	1228.63 ± 61.59	n.d.	20.15 ± 0.18 ^B^	23.85 ± 0.77 ^A^
10	Ellagic acid glucoside	PAs/HBAs	812.39 ± 28.40	n.d.	3.05 ± 0.06 ^B^	9.71 ± 1.38 ^A^
11	Vanillin	PAs/HBAs	952.01 ± 24.85	n.d.	3.72 ± 0.09 ^B^	12.89 ± 1.42 ^A^
12	Quercetin 3-*O*-glucoside	FVs/Fvols	226.31 ± 25.19	n.d.	2.25 ± 0.10 ^B^	3.57 ± 0.02 ^A^
13	Ellagic acid	PAs/HBAs	349.02 ± 16.51	n.d.	1.07 ± 0.10 ^B^	2.75 ± 0.20 ^A^
14	Quercetin 3-*O*-glucuronide	FVs/Fvols	101.10 ± 9.39	n.d.	1.61 ± 0.03 ^B^	1.93 ± 0.03 ^A^
15	Kaempferol 3-*O*-glucoside	FVs/Fvols	137.38 ± 13.05	n.d.	1.52 ± 0.0 ^A^	2.04 ± 0.18 ^A^
16	5-Sinapoylquinic acid	PAs/HCAs	89.12 ± 10.92	n.d.	2.00 ± 0.03 ^B^	2.42 ± 0.06 ^A^
17	Syringic acid	PAs/HBAs	147.44 ± 1.10	n.d.	1.61 ± 0.04 ^B^	3.40 ± 0.31 ^A^
18	Tiliroside	FVs/Fvols	239.26 ± 6.41	n.d.	1.41 ± 0.0 ^A^	1.76 ± 0.18 ^A^
	Total content		25,007.15	233.85	410.62	863.69

Data are expressed as mean ± standard deviation values of four measurements. Different uppercase letters within a row indicate significant differences between pork sausage formulations (*p* < 0.05, Tukey’s test).

**Table 8:** Content of carotenoid compounds (µg/g) in powder from rosehip waste and pork sausages.

Crt. No.	Compound	Powder From Rosehip Waste	PSc	PS2.7%rp	PS5.5%rp
1	Lutein	2.10 ± 0.21	n.d.	n.d.	n.d.
2	Lycopene	38.74 ± 0.63	n.d.	3.65 ± 0.03 ^B^	6.32 ± 0.18 ^A^
3	*β*-Carotene	77.79 ± 1.00	n.d.	1.37 ± 0.16 ^B^	3.66 ± 0.28 ^A^
	Total content	118.63	-	5.02	9.98

Data are expressed as mean ± standard deviation values of four measurements. Different uppercase letters within a row indicate significant differences between pork sausage formulations (*p* < 0.05, Tukey’s test).

## Data Availability

The original contributions presented in this study are included in this article. Further inquiries can be directed to the corresponding author.

## Abbreviations

The following abbreviations are used in this manuscript:

PSc	Control pork sausages
PS2.7%rp	Pork sausages formulated with 2.7% powder from rosehip waste
PS5.5%rp	Pork sausages formulated with 5.5% powder from rosehip waste
EHN	Easily hydrolysable nitrogen
NH_3_	Ammonia
*L**	Lightness
*a**	Redness
*b**	Yellowness
*h**	Hue angle
*C**	Chroma
Δ*E**	The total colour difference
PAs	Phenolic acids
FVs	Flavonoids
HBAs	Hydroxybenzoic acids
HCAs	Hydroxycinnamic acids
FVals	Flavanols
Fvols	Flavonols
ACNs	Anthocyanins
n.d.	Not detected
